# Objectively determined habitual physical activity in South African adolescents: the PAHL study

**DOI:** 10.1186/1471-2458-14-471

**Published:** 2014-05-19

**Authors:** Sandra N Wushe, Sarah J Moss, Makama A Monyeki

**Affiliations:** 1 Physical Activity, Sport and Recreation Research Focus Area, Faculty of Health Sciences, North-West University, Private Bag X6001, Potchefstroom 2520, South Africa

**Keywords:** Actiheart, Accelerometry, Adolescents, Habitual physical activity, South Africa

## Abstract

**Background:**

There is limited data on objectively determined habitual physical activity (PA) in 16-year old South African adolescents. The purpose of this study was to objectively determine the habitual PA of adolescents from the North West Province of South Africa by race and gender.

**Methods:**

Adolescents (137 girls, 89 boys) from the ongoing Physical Activity and Health Longitudinal Study (PAHL study), participated in the present study. Habitual PA was objectively recorded by means of the Actiheart^®^ over a period of 7 days. Time spent in moderate-to-vigorous intensity physical activity (MVPA) was assessed.

**Results:**

Average MVPA for the study sample was 50.9 ± 40.3 minutes/day. Girls were significantly more active than boys expending more time in MVPA (61.13 ± 52.2 minutes/day; *p* < 0.05) than boys (35.0 ± 32.9 minutes/day). Although white adolescents spent more time in MVPA than black adolescents, there was no significant difference in MVPA between black (47.87 ± 39.6 minutes/day; *p* = 0.58) and white adolescents (59.5 ± 41.8 minutes/day).

**Conclusion:**

Physical activity varies by both gender and race in adolescents from the North West Province of South Africa. Objectively determined data from our study indicates that girls habitually spend more time in MVPA per day than boys, and that white adolescents habitually engage in more MVPA than black adolescents. Seeing as the average MVPA per day for the entire study sample falls below the recommended daily average of 60minutes/day, adolescents should be the foremost targets of interventions aimed at enhancing habitual PA.

## Background

Adolescence is a period characterised by rapid physical growth as well as changes in physiological and behavioural patterns [1]. Recent studies in South Africa have shown unsatisfactory levels of physical activity (PA), overweight and obesity among children and adolescents [2-4]. Existing reports from nationwide cross sectional studies [3,4] and a national survey [5] indicate that only about 42% of young South Africans aged between 13–19 years participate in sufficient moderate-to vigorous physical activity (MVPA). South Africa is a country faced with the burden of a rise in non-communicable diseases of lifestyle [4], hence the low levels of adolescent PA are disturbing as there is increasing evidence that PA has an important impact on long-term health and behaviour outcomes [6,7]. It is essential to analyse patterns of habitual PA in adolescents as they serve as important functional indicators of an individual’s health status.

The most recent PA recommendation for Americans aged 6–18years is 60 minutes or more per day of MVPA [6] while other recommendations call for 30–60 min of daily MVPA [8,9]. The accurate measurement of PA in epidemiological studies still poses a major challenge in terms of feasibility and validity of the methods proposed [10]. When assessed by accelerometry, PA has been shown to decrease during adolescence [11]. After an extensive review of literature, Dumith (2011) indicated that among adolescents aged 13–19 years, the decrease in PA was higher in boys (7%) than in girls (6.3%) [12]. Very limited PA data exists for representative samples of adolescents, with even fewer objectively collected data [13], especially in Africa.

In South Africa, there are currently not many studies which objectively quantify PA in adolescents. With the exception of Cook (2008; 2012) who used pedometers [14] and uniaxial accelerometry [15] to objectively measure PA in rural black South African women, there is a dearth of information on other population groups such as adolescents. Most studies that contain important information about the habitual PA of South African adolescents have used self-report methods to assess PA [3-5,16]. The use of objective instruments to determine PA would give a much needed, clearer understanding of the habitual PA patterns of South African adolescents.

In recent years, the measurement of PA with accelerometers has become more widely used [17]. These instruments quantify body movement at 5–60 second intervals for periods of up to 21 days, thereby enabling patterns of movement or inactivity to be measured. The Actiheart*®* has been validated against the doubly labelled water method; and it emerged a technically reliable and valid tool for PA assessment in populations [18].

South Africa is a multi-racial country, where black Africans form 79.2% of the population and Caucasians form 8.9% of the population. Race is a factor that has been hypothesised to have an effect on PA. Using questionnaires, McVeigh and colleagues (2004) found significant differences in patterns of PA in 9 year old South African school children of different races [16]. White children were found to be physically more active, more likely to participate in physical education classes at school and watched less television than black children [16]. No comparable data on the racial disparity of physical inactivity and television watching among South African adolescents could be found, but it is probable that the same trend seen in 9 year old South African children as reported by McVeigh et al. (2004) could be observed in South African adolescents.

There is a need for the accumulation of accurate data with regard to objectively measuring the pattern and amount of habitual PA in South African adolescents [19]. Baseline data from the Physical Activity and Health Longitudinal Study (PAHL study) may provide valuable information on objective PA patterns of adolescents for future studies on intervention strategies. Therefore, the purpose of the study was to objectively determine habitual PA of adolescents in the North West Province of South Africa by race and gender.

## Methods

### Study design and subjects

The present study is an observational cohort study following a cross-sectional research design. The PAHL study is an observational multi-disciplinary longitudinal study that was started in 2010. The aim of the PAHL study is to describe the development of PA and determinants of health risk factors in 14 to 18 year old adolescents. More details about the study are published elsewhere [2]. The inclusion criteria for the current study was that the adolescents had to be aged 16 years in the 2012 calendar year, be of sound health and be part of the on-going PAHL study.

### Subject demographic characteristics

The Tlokwe Municipality falls within Dr Kenneth Kaunda District in the North West province of South Africa and has a population of 162,762 of which 8.7% are between the ages of 15–19 years. The Tlokwe Municipality has a population density of 61 persons/km^2^ and each household averages 3 persons. The languages predominately spoken are Setswana, Afrikaans and English. A sample of 226 adolescents (137 girls and 89 boys), participated in the study. Participant birth records were used to ascertain their age. All participants included in the current study formed part of the ongoing 5 year longitudinal PAHL study. Initially eight secondary schools from the Ikageng Township and the Potchefstroom Central town areas were randomly selected and recruited to take part in the study. Two schools from the Potchefstroom Central town areas declined to take part, and as such, six schools partook in the study. Two were from a high socio-economic area and four from the township, a low socio-economic area within the Tlokwe Local Municipality of the North West Province of South Africa. At the outset, participants who were aged 14 years and in grade 8 were purposefully selected from class lists provided so that they could be successfully followed for the duration of the 5 year PAHL study before completing high school at the age of 18 years.

### Ethical approval

Prior to the study permission to conduct the measurements was granted by the District Manager of the Department of Basic Education in Potchefstroom as well as the Ethics Committee of the Potchefstroom Campus of the North West University (Ethics number: NWU-0058-01-A1).

### Anthropometric measurements

Weight, height and skinfolds, taken on the right side of the body, were measured as described by the International Society for the Advancement of Kinanthropometry (ISAK) [20]. Weight was measured to the nearest 0.1 kilograms (kg) on a calibrated scale (Seca, Germany) with the subject standing barefoot. Triceps and subscapular skinfolds were measured on the right side of the body, to the nearest 0.1 millimetre (mm) with a skinfold calliper (Harpenden, UK) and the average of two measurements was used. Height was measured by means of a stadiometer with participants standing bare foot with their head aligned in the Frankfurt plane [20]. Variability was reduced by having all measurements taken by Level 2 ISAK anthropometrists. Intra-rater and inter-rater reliability for height, weight and skinfold measurements was not assessed. To compute percentage body fat (%BF), the internationally accepted Slaughter (1988) equation which utilises skinfolds and is approved for use in adolescents of different ethnicities and was utilised [21].

### Objectively measured physical activity (Actiheart®)

The time spent in different intensities of PA, represented in metabolic equivalents (METs), was measured by the Actiheart® (Cambridge Neurotechnology Ltd, Cambridge, UK) in 60 second epochs over seven consecutive days. Participants were instructed to wear the Actiheart® 24 hours a day, for seven days. Compliance was achieved by offering participants an incentive in exchange for wear time compliance. Data was considered valid if the participants wore the Actiheart for a minimum of 4 days, one of which was a weekend day. The average number of MET minutes/day spent in MVPA was computed by the accompanying Actiheart® software. Specific MET cut-off points describe the intensity level of PA: Sedentary (<1.5 METs); Light (1.5 METs-3METs); Moderate-to Vigorous (>3.0 METs). The particulars of the Actiheart® are described in detail elsewhere [18]. Recorded MET minutes/day of MVPA were weighed up against the current PA recommendations for adolescents. Physical activity levels (PAL), resting metabolic rate (RMR), total energy expenditure (TEE) and activity energy expenditure (AEE) in kCal/day were also measured by the Actiheart® using a set of inbuilt equations based on a branched model approach and calculated based on the combination of heart rate and accelerometry.

### Procedures

The participating schools were briefed about the purpose of the study and informed consent was given by school authorities, parents and pupils of participating schools. All measurements were performed in one day with the anthropometric and body composition measurements taken first. The Actiheart® device was placed on the chest of the participants [18] and they were instructed to wear it for 24 hours each day. After seven days the device was removed and MVPA, PAL and energy expenditure data were downloaded using the accompanying commercial software (Version 2. 132, Cambridge Neurotechnology Ltd. Cambridge, UK). The Actiheart software was used to determine the duration the device was worn. Data from adolescents who wore the device for a minimum of 4 days (one of which was weekend day) were included in the analysis.

### Statistical analysis

Data analysis was performed using SPSS Version 20 software (IBM SPSS, II). The descriptive statistics (mean and standard deviations) were performed to determine the characteristics of the participants. Independent t-tests for normally distributed data and Mann-Whitney U-test for non-normally distributed data were performed to determine differences between ethnicity and genders and calculate practical significance. A Type I error rate of *p* ≤ 0.05 was used for statistical significance.

## Results

### Subject characteristics

The gender and racial composition of the study participants is detailed in Table 1. Of the 226 participants, 137 (60.6%) were girls and 89 (39.4%) were boys. The characteristics of the participants are presented in Table 2. Body mass, stature and body mass index (BMI) differed significantly (*p* < 0.05) by race. The anthropometric data indicates that girls have significantly higher %BF (15.5 ± 5.0%; *p* < 0.05) than boys (9.3 ± 3.7%), however, there was no significant difference in %BF between black and white adolescents. Significant differences in fat mass (FM) and fat free mass (FFM) were observed between the racial groups and genders, with black adolescents recording a lower FM (7.6 ± 5.0 kg; *p* = 0.24) than white adolescents (9.4 ± 5.9 kg) and boys recording a higher FFM (54.9 ± 11.6 kg; *p* < 0.05) than the girls (48.2 ± 8.9 kg).

**Table 1:** Gender and racial composition of study participants

	**Number**	**Percentage**
**Girls**	137	60.6%
**Boys**	89	39.4%
**Black**	168	74.3%
**White**	58	25.7%
**Black girls**	110	48.6%
**Black boys**	58	25.7%
**White girls**	27	12.0%
**White boys**	31	13.7%
**TOTAL Participants**	**226**	**100%**

**Table 2:** Characteristics (mean and SD) for all subjects, stratified by gender

	**All**		**All**	
	**Boys**	**Girls**	**Black**	**White**
	**Mean ± SD**	**Mean ± SD**	**Mean ± SD**	**Mean ± SD**
Age	15.8 ± 0.7	15.7 ± 0.9	15.8 ± 0.9	15.7 ± 0.5
Stature (cm)	169 ± 0.1^a^	158 ± 0.1^a^	160.1 ± 0.1^b^	171.2 ± 0.1^b^
Body Mass (kg)	61.4 ± 15.1^a^	57.6 ± 13.2^a^	55.3 ± 11.0^b^	69.9 ± 16.3^b^
BMI (kg/m^2^)	21.1 ± 4.2^a^	22.9 ± 4.9^a^	21.7 ± 4.5^b^	23.8 ± 5.2^b^
%Body Fat	9.3 ± 3.7^a^	15.5 ± 5.0^a^	13.1 ± 5.6	12.9 ± 5.0
Fat Mass (kg)	6.1 ± 4.6^a^	9.4 ± 5.4^a^	7.6 ± 5.0^b^	9.4 ± 5.9^b^
Fat Free Mass (kg)	54.9 ± 11.6^a^	48.2 ± 8.9^a^	47.6 ± 7.8^b^	60.3 ± 11.9^b^
	**Boys**		**Girls**	
	**Black**	**White**	**Black**	**White**
	**Mean ± SD**	**Mean ± SD**	**Mean ± SD**	**Mean ± SD**
Age	15.84 ± 0.8	15.65 ± 0.1	15.7 ± 0.5	15.7 ± 0.5
Stature (cm)	166.1 ± 0.1	177.2 ± 0.1	157.2 ± 0.1	165.1 ± 0.1
Body Mass (kg)	54.5 ± 8.7	74.2 ± 16.2	55.8 ± 12.1	64.9 ± 15.2
BMI (kg/m^2^)	19.8 ± 0.8	23.7 ± 5.0	22.7 ± 4.8	24.0 ± 5.6
%Body Fat	8.5 ± 2.3	10.8 ± 5.1	15.5 ± 5.4	15.5 ± 3.5
Fat Mass (kg)	4.7 ± 1.9	8.7 ± 6.6	9.1 ± 5.4	10.4 ± 4.9
Fat Free Mass (kg)	49.4 ± 7.5	65.5 ± 10.6	46.7 ± 7.8	54.5 ± 10.6
White				

RMR = resting metabolic rate; n = number; %BF = percentage body fat; ± = standard deviation. Values represented as means ± standard deviation. Comparisons between gender and race groups were completed using independent t-tests when data was normally distributed and Mann-Whitney U-test for non-normally distributed data.

^a^*p* < 0.05 Girls vs Boys.

^b^*p* < 0.05 Black vs White.

### Energy expenditure: gender differences

Means and standard deviations of the participants’ average daily energy expenditure are shown in Table 3. There are marked differences between race and gender groupings in all aspects of energy expenditure and PA. Boys (2459 ± 289 kCal/day; *p* < 0.05) showed significantly higher TEE than girls (2229 ± 316 kCal). The girls AEE (589.7 ± 225 kCal/day) was significantly (*p* < 0.05) higher than that of the boys (477.1 ± 160 kCal/day).

**Table 3:** Means and standard deviations (SD) for average daily energy expenditure by gender and race

	**All**		**All**	
	**Boys**	**Girls**	**Black**	**White**
	**Mean ± SD**	**Mean ± SD**	**Mean ± SD**	**Mean ± SD**
TEE(kCal/day)	2459.0 ± 380.6^a^	2229.0 ± 352.1^a^	2247.0 ± 310.3^b^	2534 ± 474.2^b^
AEE (kCal/day)	477.1 ± 160.1^a^	589.7 ± 225.0^a^	550 .1 ± 194.2^b^	530.7 ± 248.9^b^
Mean Weekday TEE (kCal/day)	2482.0 ± 400.4^a^	2255.0 ± 385.5^a^	2270.0 ± 339.9^b^	2559.0 ± 498.9^b^
Mean Weekend TEE (kCal/day)	2383.1 ± 400.9^a^	2161.0 ± 356.2^a^	2188.1 ± 349.3^b^	2424.1 ± 444.7^b^
PAL	1.41 ± 0.09^a^	1.57 ± 0.15^a^	1.53 ± 0.14^b^	1.45 ± 0.16^b^
	**Boys**		**Girls**	
	**Black**	**White**	**Black**	**White**
	**Mean ± SD**	**Mean ± SD**	**Mean ± SD**	**Mean ± SD**
TEE(kCal/day)	2317.0 ± 288.7	2725.0 ± 392.9	2209.0 ± 316.0	2314.0 ± 469.8
AEE (kCal/day)	469.8 ± 152.0	491.0 ± 177.8	593.1 ± 201.3	577.1 ± 308.6
Mean Weekday TEE (kCal/day)	2323.0 ± 291.9	2779.0 ± 409.0	2243.0 ± 360.9	2306.1 ± 477.3
Mean Weekend TEE (kCal/day)	2256.0 ± 347.6	2620.1 ± 390.8	2152.1 ± 346.5	2199.0 ± 398.3
PAL	1.73 ± 0.09	1.39 ± 0.09	1.58 ± 0.14	1.53 ± 0.18

n = number; TEE = total energy expenditure; AEE = active energy expenditure; PA = physical activity.

Values represented as means ± standard deviation. Comparisons between gender and race groups were completed using independent t-tests when data was normally distributed and Mann-Whitney U-test for non-normally distributed data.

^a^*p* < 0.05 Girls vs Boys.

^b^*p* < 0.05 Black vs White.

### Energy expenditure: ethnic differences

White boys (2725 ± 392 kCal/day) recorded the highest TEE followed by black boys (2317 ± 288 kCal/day). Black girls recorded the lowest TEE (2209 ± 316 kCal/day) of all participants. The same trend was observed for average weekday and average weekend TEE with black adolescents recording a lower average weekday (*p* < 0.05) and weekend TEE (*p* < 0.05) than white adolescents.

### Physical activity levels (PAL)

Physical activity level (PAL) is calculated by dividing TEE by RMR. Girls profiled a significantly higher PAL (1.57 ± 0.15; *p* < 0.05) than boys (1.41 ± 0.10). Additionally, black adolescents (1.53 ± 0.14; *p* < 0.05) recorded significantly higher PAL than white adolescents (1.45 ± 0.16).

### Duration of physical activity at various intensities

Table 4 shows the results of the time spent (minutes/day) by the participants in habitual PA of different intensity. In the present study, girls (61.13 ± 52.2 minutes/day; *p* < 0.05) spent more time in MVPA per day than boys (35 ± 32.9 minutes/day). There was no significant difference in the MVPA of black (47.87 ± 39.6 minutes/day; *p* = 0.058) and white adolescents (59.50 ± 41.82 minutes/day).

**Table 4:** Daily physical activity (MET minutes/day) at different intensity levels for boys and girls

	**All**		**All**	
	**Boys**	**Girls**	**Black**	**White**
	**Mean ± SD**	**Mean ± SD**	**Mean ± SD**	**Mean ± SD**
Sleep/Sedentary PA < 1.5 METs	1178.0 ± 88.9^a^	1070.0 ± 108.4^a^	1091.0 ± 103.1	1174.0 ± 122.3^b^
Light 1.5 – 3.0 METs	234.1 ± 81.7^a^	306.9 ± 85.5^a^	296.0 ± 80.47	225.0 ± 99.5^b^
Moderate PA >3 – 6 METs	33.2 ± 32.5^a^	58.6 ± 39.0^a^	46.2 ± 37.6	55.6 ± 40.8
Vigorous PA >6 METs	1.8 ± 2.7	2.5 ± 4.9	1.7 ± 3.2	3.9 ± 5.8^b^
MVPA >3 METSs	35.0 ± 32.9^a^	61.1 ± 52.2^a^	47.9 ± 39.6	59.5 ± 41.8
	**Boys**		**Girls**	
	**Black**	**White**	**Black**	**White**
	**Mean ± SD**	**Mean ± SD**	**Mean ± SD**	**Mean ± SD**
Sleep/Sedentary PA < 1.5 METs	1154.0 ± 85.1	1224.0 ± 78.6	1058.0 ± 96.48	1118.0 ± 139.5
Light 1.5 – 3.0 METs	259.0 ± 78.54	187.0 ± 65.5	316.3 ± 74.6	269.0 ± 114.1
Moderate PA >3 – 6 METs	25.9 ± 24.6	46.7 ± 40.7	56.9 ± 38.9	65.8 ± 39.1
Vigorous PA >6 METs	1.2 ± 1.7	2.8 ± 3.8	1.8 ± 3.9	5.1 ± 7.5
MVPA >3 METSs	27.2 ± 25.3	49.6 ± 40.5	58.8 ± 41.4	70.9 ± 41.1

METs = metabolic equivalents; PA = physical activity; MVPA = moderate-to-vigorous physical activity.

Values represented as means ± standard deviation. Comparisons between gender and race groups were completed using independent t-tests when data was normally distributed and Mann-Whitney U-test for non-normally distributed data.

^a^*p* < 0.05 Girls vs Boys.

^b^*p* < 0.05 Black vs White.

### Minutes of MVPA per day

**Figure 1:**
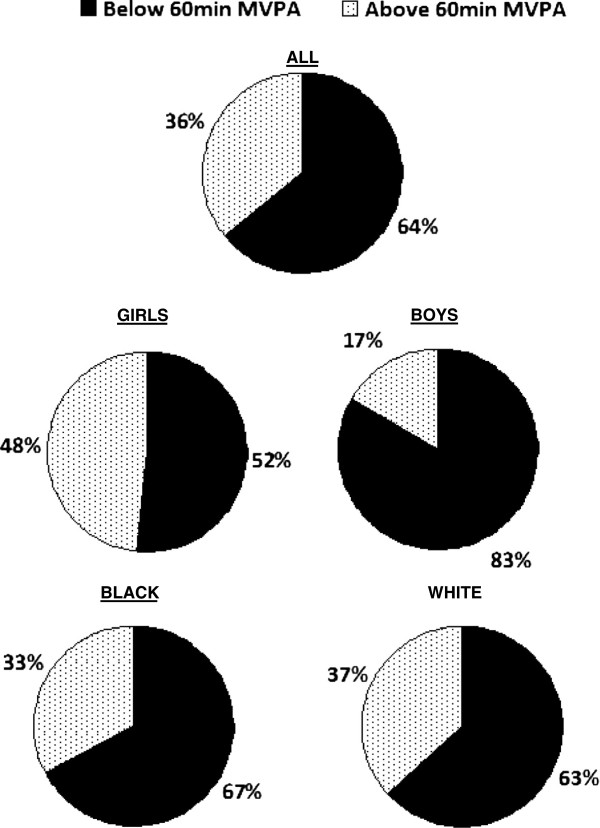
Representation of the participants who meet the recommendation of 60min daily MVPA.

The compliance of adolescents in this study to the recommendation of 60min/day of MVPA (Figure 1) indicates that only 36% of the adolescents from the Tlokwe Municipality of the North West Province of South Africa attained 60min/day of MVPA.

## Discussion

The present study provides data on objectively measured habitual PA of adolescents from the Tlokwe Municipality of the North West Province of South Africa. PA was determined using a combined accelerometer and heart rate monitor, the Actiheart®. One of the most compelling findings of our study is its consistency with the international phenomenon of low PA in adolescents [22,23]. Up until now, most of the studies on habitual PA in South Africa were done using questionnaires [4,24]. However, pedometers [14] and accelerometry [15] have been used to assess PA in rural black female South Africans as well as 8–12 year old South African children from disadvantaged schools [25]. The implication of our study is of great consequence, as to our knowledge, this study is the first to report objectively measured PA over a period of seven days, using the Actiheart® in a large number of South African adolescents.

The adverse effects of physical inactivity in adolescents have been reported to be related to the development of cardiovascular risk factors which lead to mortality [4,6]. Consistent with the accelerometry determined PA findings of Americans aged 12–15years [26] and Thai adolescents aged 13–19years [27], our study reported that over 60% of South African adolescents are not sufficiently active. This means that less than 40% of adolescents from the North West province of South Africa meet the recommendation of 60 minutes of MVPA per day and are at risk of obesity, hypertension and diabetes. Despite our sample being less representative than the 2012 South African National Health and Nutrition Examination Survey (SANHANES) [4], the low level of PA among South African adolescents from the Tlokwe Municipality of the North West Province is remains evident.

Our findings indicate that on average, South African adolescents are more active on weekdays than weekends irrespective of gender and race. Our findings are in agreement with Compte (2013), who used accelerometry to determine MVPA in 12–15 year old Canadian adolescents and reported that similarly, they spent more minutes/day in MVPA on weekdays than on weekends irrespective of gender [28]. Likewise, using accelerometry, Konharn (2012) also reported more activity on weekdays than on weekends in 13–18 year old Thai adolescents [27]. With reference to South African adolescents from the Tlokwe Municipality of the North West Province, it is possible that walking to school (Non Exercise Activity Thermogenesis–NEAT transport domain) [29] or school related physical education/scheduled sport is the reason behind higher weekday MVPA. Conversely, in 2008, Mamabolo and colleagues revealed that ±14½ year old township adolescents residing in the North West Province of South Africa were more active on weekends than on weekdays [24]. This disparity is likely due to the fact that Mamabolo and colleagues used questionnaires and not objective means to asses PA. The homogeneity of our findings with those of Konharn [27] and Compte [28] highlights the need to take into account weekday and weekend differences when developing PA interventions for adolescents.

Habitual PA influences a large portion of energy expenditure. In addition to being represented in terms of minutes/day of MVPA, physical activity and can also be articulated as a calculated PAL value or measured AEE value. The higher AEE observed among girls in the present study may be an indirect derivative of their higher FM which has been shown to also influence energy expenditure. Our findings are in agreement with Livingstone (1992) who, using indirect calorimetry, reported higher PAL values in 15 year old girls than boys from Northern Ireland [30]. Several years later, using 15 year old Swedish adolescents, Bratteby (1998) gave disparate findings as boys recorded higher PAL values than girls [31]. Since PAL is calculated by dividing TEE by RMR, it is evident that RMR is a critical factor in the determination of PAL. The peculiarity of the results of the current study may be attributed to the dissimilar ratio of measured TEE/RMR between the girls and the boys. It is clear that contemporary studies investigating the PAL and AEE of adolescents need to be done as reference data is essential for a better understanding of the effect of PA on adolescent growth, health and development.

It is apparent that the recommendation of 60 minutes/day of MVPA is currently not being met and this has potentially negative implications related to the development of cardiovascular risk factors. Our findings concur with those of Collings (2014), who using accelerometry, determined that United Kingdom adolescents (15 years) spent only 41.1 minutes/day in MVPA [22]. Our results, however, provide more relevant race specific data and give a better profile of habitual PA of black and white adolescents from the Tlokwe Municipality of the North West Province as physical inactivity in South Africa is often associated with poor, disadvantaged, black township communities [25]. A 2005 study by Miller et al. suggested that girls are more likely than boys to participate in moderate activity only and not in vigorous activity [32]. However, the main limitations of Miller’s report are that PA was assessed by questionnaire and not objectively, the second is that an American college-aged cohort was used.

### Limitations

One limitation of the present study, which should be noted when interpreting the findings, is its cross-sectional nature, which limits data to one point in time. A longitudinal design would be more advantageous in terms of providing more definitive conclusions. In addition, the fact that a 60 second epoch were used is a limitation on the accuracy of the minutes/day of MVPA as literature prescribes that lower epochs of 5, 10, 15, 30 seconds be used in order to maximise accuracy. Lastly, although representative of the racial composition of the South African population, the low number of white adolescents compared to black adolescents in our study may be viewed as a limitation. Besides the limitations, it is interesting to note that this study managed to objectively measure PA in a large number of South African adolescents. Instruments such the Actiheart® can provide PA data at a level of accuracy and specificity that has not been achieved before in South Africa.

## Conclusion

PA varies by both gender and race in adolescents from the North West Province of South Africa. Objectively determined data from our study indicates that girls habitually spend more time in MVPA per day than boys, and that white adolescents habitually engage in more MVPA than black adolescents. Given the fact that the study sample did not meet the recommended daily PA guideline, urgent strategies to inculcate the culture of regular PA as a preventative measure of chronic diseases of life style are needed. Adolescents should be the foremost targets of interventions aimed at enhancing habitual PA.

## Abbreviations

AEE: Activity energy expenditure; %BF: Percentage body fat; BMI: Body mass index; FM: Fat mass; FFM: Fat free mass; ISAK: International society for the advancement of kinanthropometry; Kg: Kilo grams; mm: Millimetres; METs: Metabolic equivalents; MVPA: Moderate-to-vigorous physical activity; NEAT: Non-exercise activity thermogenesis; PA: Physical activity; PAHL study: Physical activity and health longitudinal study; PAL: Physical activity levels; RMR: Resting metabolic rate; SANHANES: South African national health and nutritional examination survey; TEE: Total energy expenditure.

## Competing interests

The authors declare that they have no competing interests.

## Authors’ contributions

SNW designed the cross sectional study, performed data collection and wrote the paper. SJM assisted in data collection, provided guidance with statistical analysis and write up of the paper and gave critical review input in the manuscript. MAM is the principal investigator of the PAHL study, performed data collection, assisted in statistical analysis and the write up of the paper. All the authors participated in the review of the manuscript, read and approved the final manuscript.

## Pre-publication history

The pre-publication history for this paper can be accessed here:

http://www.biomedcentral.com/1471-2458/14/471/prepub
